# Relationships between the Perception of Footwear Comfort and the Fear of Falls in People at the Early Period of Old Age

**DOI:** 10.3390/ijerph19106267

**Published:** 2022-05-21

**Authors:** Ewa Puszczalowska-Lizis, Wioletta Mikulakova, Sabina Lizis, Karolina Koziol, Jaroslaw Omorczyk

**Affiliations:** 1 Institute of Health Sciences, Medical College, University of Rzeszow, Warzywna 1a, 35-959 Rzeszow, Poland; sabina.lizis92@gmail.com; 2 Department of Physiotherapy, Faculty of Health Care, University of Prešov, Partizánska 1, 080 01 Prešov, Slovakia; wioletta.mikulakova@unipo.sk; 3 Health Center Tuchow, Szpitalna, 33-170 Tuchow, Poland; karolina.koziol25@gmail.com; 4 Faculty of Physical Education and Sports, Institute of Sport, University School of Physical Education, John Paul II 78, 31-571 Krakow, Poland; jarekomo@interia.pl

**Keywords:** footwear, falls, health promotion, public health, diagnosis, treatment

## Abstract

Objective: The present study aimed to analyze the relationships between the perceptions of footwear comfort with fear of falls in younger-old women and men. Participants: the population sample involved 100 free-living community dwellers aged 65–74. Design: the Falls Efficacy Scale-International and a visual analogue scale to assess perception of footwear comfort were used as research tools. Results: there were statistically significant differences in the FES-I results in people who suffered a fall in the last year compared to those who did not experience a fall (*p* < 0.001), as well as in the subjective assessment of mediolateral control in people who have suffered and have not suffered a fall in the last year (*p* = 0.033). In women, statistically significant relationships were found in the subjective assessment of shoe comfort in terms of arch height (*p* = 0.025) and material properties of the footwear (*p* = 0.036) with the results of FES-I. Conclusions: People who have fallen show a higher level of fear of falling. The assessment of footwear comfort in terms of mediolateral control was lower in the younger-old who had experienced a fall in the last year. In women, a worse assessment of arch height and material properties of the footwear is accompanied by greater fear of falling.

## 1. Introduction

Falls in the elderly, defined as incidents during which a person unintentionally lands on the floor or other low-lying surface, are the result of a gradual deterioration in health and functional capacity due to the process of involution [1,2,3]. They are one of the main causes of injuries leading to immobilization and, consequently, secondary changes such as deep vein thrombosis, pressure ulcers, inflammation of the lungs and urinary tract, hypothermia, and joint contractures [4]. One of the consequences may also be the fear of subsequent falls, which is a specific type of anxiety. On the one hand, it causes fear of physical injury, long-term damage to health, and disability, while on the other, it is a source of shame, loss of self-confidence, and a sense of dependence on other people. Consequently, activity in everyday life is limited and the quality of life deteriorates [5,6,7]. 

Decrease in muscle strength due to the loss of motor neurons, muscle fibers, and aerobic capacity causes gait adaptations in elderly people. These changes are manifested by a reduction in gait speed, less extension in the hip and knee joints, reduced ankle dorsiflexion angle at heel-strike, decreased step and stride length, and altered step width. What is also observed is increased double-support time, stance time, and quadriceps energy absorption, and a reduction in power during toe-off are also observed [8]. 

Improper footwear, i.e., poorly fitted, excessively worn or with structural defects in the form of poor heel or the upper, or insufficient heel stiffening, may play a role in increasing the risk of falls in the elderly [9]. Additionally, footwear with elevated or narrow heels, soft midsoles, and lack of fixation is considered to be detrimental [10]. Wearing such footwear can significantly increase the sway of the body’s center of gravity, predisposing to loss of balance and, consequently, falls [11,12,13,14,15].

Therefore, entering into the early period of old age should be the moment that initiates changes in the preferences of footwear. This is especially true of women, who should decide to change their past habits when they wore high-heeled shoes with narrow toes [16,17]. In order to neutralize the effects of progressive involutional changes and maintain the optimal—in relation to age—level of quality of life and functional fitness, the younger-old should be guided by functionality and comfort instead of fashion or aesthetic considerations in the decision to choose footwear [18,19,20,21,22]. 

Data in the literature show that many elderly people wear inappropriate footwear that is excessively flexible, has thick, soft midsoles, smooth outsoles, and/or is too long and wide. Shoes are replaced infrequently, possibly due to a lack of knowledge of the characteristics of the respective shoes and/or for financial reasons [9,11,12,19]. This is why we proposed to seniors to test a specific model of shoes of a selected brand, in terms of their usefulness in everyday life. 

The aim of the study was to analyze the relationships between the perceptions of comfort of footwear specific for gender of a certain brand with fear of falls in younger-old women and men. The questions included in the study were as follows:

1. Does gender and the fact of having a history of a fall in the last year differentiate the fear of fall in the younger-old?
2. Does the fact of suffering a fall within the last year differentiate the subjective assessment of the comfort of shoes in younger-old?
3. What are the relationships between the subjective perception of footwear comfort and FES-I scale results in the younger-old women and men?

## 2. Materials and Methods

### 2.1. Participants

The study group was selected randomly using dependent simple sampling (without replacement) from individuals over the age of 65, who were inhabitants of a purpose-built housing estate for seniors. The inclusion criteria were as follows: age within the range of 65 to 74; being a resident of a housing estate for the seniors; physical capacity to walk without orthopaedic aids; wearing footwear specific for gender of a certain brand for 7 days before the survey, for a minimum of seven hours a day; expressing written informed consent to take part in the study. On the other hand, any severe form of cognitive impairment, any neurological conditions, alcohol addiction, regular administration of medications causing impairment of an individual’s sense of balance (e.g., antidepressants, antipsychotics, addiction to sedatives and/or sleep-inducing medications), any type of lower limb amputations, first aid dressings/orthoses, recent injuries within the lower extremity or any foot bones problems, wounds, feet ulceration, and painful toe deformation (e.g., hammer toes) were exclusion criteria from the study.

### 2.2. Design

The cross-sectional study was conducted in April 2021. Initially, the respondents filled in a form containing questions on basic sociodemographic data and information on falls. 

The fear of falls was assessed in the surveyed seniors with Falls Efficacy Scale-International, FES-I [23] from a Polish translation by Zak et al. [6]. FES-I includes 16 items with a high degree of reliability (ICC = 0.960 [23]). The responses were comprised in a 4-point scale (score in the range of 1–4 points for each item). Respective domain scores were scaled in a negative direction: 1—“not at all concerned”, 2—“somewhat concerned”, 3—“fairly concerned”, 4—“very concerned”. The score was the sum of points ranging from 16 (no anxiety) to 64 (strong anxiety). 

The subjects were handed the study questionnaires during meetings at the senior club. All respondents were instructed on how to complete the questionnaires and hand them over after answering the questions.

A visual analogue scale (VAS) was applied to evaluate the perception of footwear comfort, as it is believed to be a reliable tool to assess subjective perception of footwear, as ICC = 0.799 [24]. Prior to the assessment of the footwear comfort perception, the study group had been wearing the footwear for twenty minutes; next, they were asked to walk a distance of 10 m at a comfortable speed.

The seniors evaluated such aspects of the footwear concerning its perceived comfort, such as: shoe length, shoe forefoot width (width of the shoe in the forefoot region), shoe heel width (width of the shoe in the heel region), heel height (height at which the hindfoot is raised in relation to the forefoot), heel cushioning (softness/hardness of the midsole in the heel region), forefoot cushioning (softness/hardness of the midsole in the forefoot region), arch height (medial arch height of the insole), mediolateral control (position of the foot controlled by the shoe), overall comfort: overall impression of the shoe [24,25]. Moreover, a structural element of the shoes was evaluated, which had an impact on comfort perception, “material properties of the footwear”, which is connected with the quality/number of materials used and sewing type.

The scale of comfort applied in the present study was a line of 100 mm in length with “not comfortable at all” (0 comfort score) on the left and “most comfortable” (10 comfort score) on the right [24]. 

**Figure 1:**
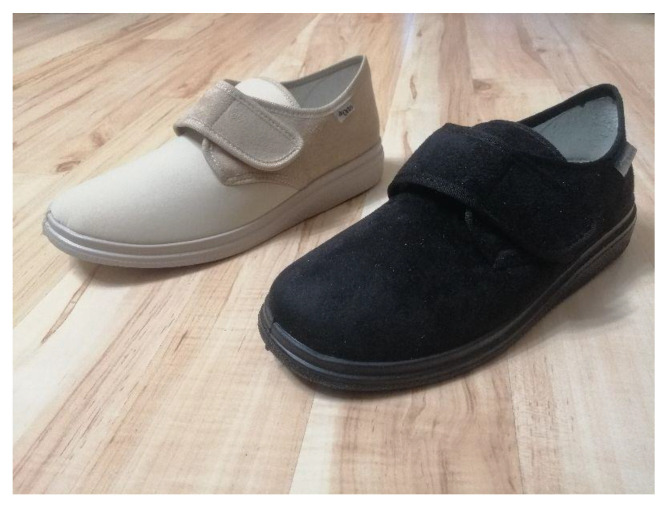
Befado Dr orto footwear used in the study.

The evaluation included gender-specific footwear of a specific brand (Befado Dr orto) worn for 7 days before the study, for at least 7 h a day. Therefore, the respondents had the opportunity to test the shoes in various situations, such as indoor vs. outdoor, on a smooth surface vs. uneven surface, while walking on a flat surface vs. while walking up the stairs, etc. The shoes were purchased by the subjects and of the right size and fitted to the foot length. In order to select the appropriate shoe size, the SULPO measure (Insoles.pl; catalogue number: D085) was used. The measure was a plate equipped with a scale and a movable slider to facilitate reading the results. The plate ended at the rear with a vertical wall (limiter). In order to determine the length of the foot, the subject, with the help of the examiner, placed it on the plate so that the heel rested against the limiter, and then the examiner adjusted the slider to the longest toe. Note that the longest toe was not necessarily the first toe. Participants stood barefoot and relaxed, with their feet slightly apart and with the weight evenly distributed between both feet. The number in the center of the slider window indicated the foot length in centimeters. The same protocol was established for the other foot. The larger of the two sizes was recorded as the participant’s shoe size. Then, a selection of footwear corresponding to the length of the insole was made according to the size table. After buying shoes, before the test, the researchers additionally examined the adequate adjustment of the shoes to the participants feet in an even weight-bearing standing position. The footwear was fitted when the toes could move freely, while the heel had a secure position at the heel counter. The credibility of the subjects’ participation in the 7-day test of footwear was acknowledged based on their declaration. Moreover, the footwear wear condition was inspected during the tests. The same model of footwear was worn by all subjects, with beige shoes for women: catalogue number: 036D005, and black ones for men: catalogue number: 036M007 (Figure 1). 

The particular model of footwear was chosen due to a relatively good relation between price and quality, especially in terms of functional and health features. The footwear had wide shoe forefoot, a stiffened shoe heel with a profiled insole, and a shock-absorbing non-slipping sole. The upper was manufactured from a flexible, stretchable fabric. The Velcro fastening did not hinder flexing and adjusted to the foot’s thickness. The inner part was made of a soft “Silber (Ag)” material manufactured using a special technology with silver ions.

The respondents evaluated footwear comfort in the presence of the researcher, after thorough explanations about the assessed footwear parts/aspects. The participants were given detailed instructions on answer marking on the visual analogue scale. Additional explanations were given if necessary. The Bioethics Committee of the University of Rzeszow approved the study (Approval Ref. No. 4/04/2020). The study was conducted in line with the Declaration of Helsinki.

### 2.3. Statistical Analysis

Normal distribution of variables was verified by means of the Shapiro–Wilk test. The dependences between gender and participant characteristics with data on falls were tested using the Pearson Chi-square test. The assessment of inter-groups differences in the values of FES-I in groups distinguished by gender and the fact of suffering a fall within the last year, as well as the perception of footwear comfort in people who have and have not suffered a fall in the last year, was performed using the Mann–Whitney U test. Spearman’s rank correlation was used to analyze the relationships between the subjective assessment of footwear comfort and the values of FES-I. The statistical significance was set at *p* < 0.05. The Stat Soft STATISTICA application (ver. 13.1 PL; StatSoft Inc., Tulsa, OK, USA; StatSoft, Krakow, Poland) was used to process all test results.

## 3. Results

The population sample involved 100 free-living community dwellers aged 65–74 years (younger-old), including 50 women ($\bar{x}$ = 69.62 ± 3.29 years) and 50 men ($\bar{x}$ = 69.82 ± 3.01 years). Table 1 presents the housing situation of the surveyed women and men. Statistically significant relationships were found between the sex of the respondents and their housing situation. Men almost twice as often as women declared that they lived alone (*p* = 0.021).

The data in Table 2 indicate lack of statistically significant relationship between the gender of the studied seniors and the variables related to falls.

The data in Table 3 show that people who suffered a fall in the last year had higher scores in the FES-I compared to those who did not experience a fall (*p* < 0.001).

The data in Table 4 indicate statistically significant differences in the subjective assessment of mediolateral control in people who have suffered and have not suffered a fall in the last year (*p* = 0.033). The values defining this feature of footwear were lower in the case of people who had suffered a fall.

In women, statistically significant relationships were found in the subjective assessment of shoe comfort in terms of arch height and material properties of the footwear with the results of FES-I. A negative direction of these relationships indicates that lower assessment of footwear comfort in terms of medial arch height of the insole was accompanied with higher FES-I score (*p* = 0.025), while lower assessment of material properties of the footwear (*p* = 0.036) were accompanied by higher FES-I scores (Table 5).

## 4. Discussion

In our material, gender was not a factor differentiating the level of fear of falling, while people who suffered a fall during the last year showed a higher level of fear of falling compared to those who did not fall. More than half of the surveyed women and men showed a reduction in their own activity in everyday life as a result of a fall. These results can be explained as a consequence of mental stress. Denkinger et al. [26] called this condition “post-fall syndrome”, which is a potential, modifiable threat to the autonomy and quality of life of the elderly. This is in line with the results of the study by Hornyak et al. [27] in group community-dwelling subjects over the age of 64, who were independent in ambulation with or without an assistive device. Fear-induced avoidance of activity worsened functional fitness and activity in everyday life. Additionally, Choi et al. [28], in a study of elderly women in Korea, concluded that longer exposure to fear of falling was associated with an increased risk of functional decline.

Hatton et al. [29] emphasized the importance of footwear for maintaining balance and gait pattern in the elderly. According to the authors, the use of properly selected footwear has an impact on the improvement of the foot position and distribution of pressure forces on the sole surface, improved motor control, and gait kinematics, as well as the proximal alignment of the lower limbs, shock absorption, and alleviation of foot pain. Burke [30] pointed out that footwear comfort perception may impact the neuromuscular control of challenging balance tasks. According to Matthias et al. [31], perceived footwear comfort influences wearability and can impact on physical mobility, performance, and foot-related complaints. Our study shows that the fact of experiencing a fall differentiates the subjective assessment of shoe comfort in terms of mediolateral control. This rating was lower in the younger-old who had suffered a fall within the last year. These data may suggest that people with a higher level of fear of falls have more difficulty in achieving mediolateral stability compared to non-fallers. This suggests that they may need additional stabilizing insoles in order to alter the strategies that control static balance and dynamic movement patterns. Palluel et al. [32], in a study on the effect of using insoles covered with semi-rigid plastic spikes, observed a reduction in the deviations of the center of foot pressure, both in the anteroposterior and mediolateral directions in elderly people. This suggests a greater balance control in a free-standing position as a result of the use of this type of insole. Similarly, the research by Huang et al. [33] showed that footwear with nodulous insoles ensures tactile stimulation to plantar mechanoreceptors that could elicit functional benefits on balance control in seniors.

It is worth emphasizing here that the subjective assessment of the perception of footwear comfort in the other aspects did not differentiate between people who experienced falls and non-fallers, and relatively high average values of comfort scores with regard to individual aspects of footwear comfort led to the conclusion that the subjective assessment of the perception of footwear comfort of the respondents is high enough for this type of shoes to be recommended to seniors.

An interesting issue is the relationship between the subjective assessment of footwear comfort and the results of the FES-I. In our own material, younger-old women’s subjective perception of footwear comfort regarding arch height and the material properties of the footwear shows correlation with FES-I scores. A worse assessment of the aforementioned features of shoes is accompanied by a greater fear of falling. Though the intergender comparison of the FES-I results with the Mann–Whitney U test did not show statistically significant differences, the Spearman test results showed statistically significant relationships between the variables: arch height and material properties, related to the perception of footwear comfort with the results of the FES-I, compared to lack of similar relationships in men. Doubtless this is due to the fact that the intergender comparison concerns analyzes on averaged (rank) values, while the correlation analyzes concern the relationships between two variables separately in each of the respondents. Therefore, despite the lack of statistically significant inter-gender differences in the FES-I values, in women the variables related to the perception of footwear comfort correlated with the FES-I results, while no relationships were found in men. Our research is pioneering in terms of the relationship between the perception of footwear comfort and the fear of falls in people in old age, hence it is difficult to relate our results to the studies of other authors. It seems, however, that the occurrence of the above-mentioned relations in women and the lack of any connections in men may be dictated by a more intense decline of proprioception in the female gender. Cooper et al. [34] emphasized that a relatively faster reduction of proprioception, which was the cause of worse stability in older women, results from a faster decrease in muscle strength compared to men. In turn, according to Menant et al. [8] and Cronin et al. [17], the causes of proprioception disorders in older women are the result of the past wearing of less-comfortable shoes, which are often too tight, stiff, and have high heels.

Summarizing the results of our study, it can be concluded that the features determining the comfort of footwear can be a useful guide in the selection of shoes for seniors. It seems that footwear with a closed heel, Velcro fastening, and a stable sole appear to be comfortable, and wearing them may reduce the risk of falls in elderly people. Therefore, consideration should be given to including these types of shoes for use as part of a multi-faceted fall prevention program. Our research is the starting point for further issues, such as neurophysiological reactions to the condition of shoe profiling. This is particularly important for improving lateral stability during free standing and locomotion. Such management is clinically important, especially in view of the relationship between lateral stability and falls in the elderly.

## 5. Conclusions

1. People who have fallen in the last year show a higher level of fear of falling. Gender is not a factor differentiating fall anxiety levels in the younger-old.
2. The fact of experiencing a fall differentiates the subjective assessment of the comfort of the footwear in terms of mediolateral control. The assessment was lower in the younger-old who had experienced a fall in the last year.
3. In younger-old women, subjective perception of footwear comfort in terms of arch height and material properties of the footwear shows a relationship with the results of FES-I. Worse assessment of arch height and material properties of the footwear is accompanied by greater fear of falling. In the case of men, no statistically significant relationships were found.

### Study Limitations

The main limitations of our study consisted of the fact that many factors will affect falls in the elderly, such as auditory–visual deficiency, physical activity, foot morphology and alignment, balance, temporospatial gait parameters, and comorbidity, which makes it rather hard to select the specific variables that may impact the fear of falls independently. Consequently, each one of the variables not allowed for in the assessment may well become a confounding variable, which ultimately may impact the final conclusions. Despite these limitations, the results bring significant implications for people at the early period of old age.

## Figures and Tables

**Table 1:** Housing situation of the participants.

Variable	Women	Men	Chi-Square Test
Housing situation, *n* (%)			
Lives alone	12 (24.0)	23 (46.0)	χ^2^(1) = 5.32; *p* = 0.021 *
Lives with the family	38 (76.0)	27 (54.0)	

Abbreviations: *n*—number of subjects; %—percent of subjects; χ^2^—value of the Chi-square test statistic; *p*—probability value. Notes: * *p* < 0.05.

**Table 2:** Characteristics of the falls experienced by the respondents.

Variable	Women	Men	Chi-Square Test
Falls in the past year, *n* (%)			
Yes	27 (54.0)	28 (56.0)	χ^2^(1) = 0.04 *p* = 0.840
No	23 (46.0)	22 (44.0)	
Number of falls during the last year, *n* (%)			
1 fall	8 (30.0)	4 (14.0)	χ^2^(5) = 4.97 *p* = 0.419
2 falls	8 (30.0)	9 (33.0)	
3 falls	6 (22.0)	4 (14.0)	
4 falls	3 (11.0)	6 (21.0)	
5 falls	2 (7.0)	3 (11.0)	
6 or more falls	0 (0.0)	2 (7.0)	
Falls have occurred, *n* (%)			
Indoor	13 (48.0)	16 (57.0)	χ^2^(1) = 0.45 *p* = 0.504
Outdoor	14 (52.0)	12 (43.0)	
Direction of falls of the surveyed people, *n* (%)			
Forward	4 (15.0)	6 (21.0)	χ^2^(2) = 1.48 *p* = 0.478
Backwards	9 (33.0)	12 (43.0)	
Sidewards	14 (52.0)	10 (36.0)	
Need for treatment after a fall, *n* (%)			
Yes	8 (30.0)	14 (50.0)	χ^2^(1) = 2.37 *p* = 0.123
No	19 (70.0)	14 (50.0)	
The need for immobilization after a fall, *n* (%)			
Yes	4 (15.0)	9 (32.0)	χ^2^(1) = 2.28 *p* = 0.130
No	23 (85.0)	19 (68.0)	
Limitation of own activity in everyday life as a result of falling, *n* (%)			
Yes	15 (56.0)	16 (57.0)	χ^2^(1) = 0.01 *p* = 0.905
No	12 (44.0)	12 (43.0)	

Abbreviations: *n*—number of subjects; %—percent of subjects; χ^2^—value of the Chi-square test statistic; *p*—probability value. Notes: * *p* < 0.05.

**Table 3:** Comparison of the FES-I values in the groups by gender and the fact of suffering a fall in the last year.

$\bar{x}$ ± SD	Max-Min	Me	$\bar{x}$ ± SD	Max-Min	Me	Mann-Whitney U Test
**Women (*n* = 50)**			**Men (*n* = 50)**			
37.50 ± 9.63	60.00–20.00	36.50	40.88 ± 9.80	60.00–23.00	41.50	Z = −1.77; *p* = 0.076
Fallers (*n* = 55)			Non-fallers (*n* = 45)			
45.15 ± 7.36	60.00–28.00	44.00	31.91 ± 7.18	57.00–20.00	39.00	Z = 7.11; *p* < 0.001 *

Abbreviations: $\bar{x}$—arithmetical average value; SD—standard deviation; max—maximum value; min—minimum value; Me—median; Z—value of the Mann–Whitney U test statistic; *p*—probability value. Notes: * *p* < 0.05.

**Table 4:** Subjective assessment of the comfort of the footwear in people who have and have not suffered a fall within the last year.

Variable	Fallers			Non-Fallers			Mann-Whitney U Test
	$\bar{x}$ ± SD	Max-Min	Me	$\bar{x}$ ± SD	Max-Min	Me	
Shoe length	8.51 ± 1.54	10.00–3.00	9.00	8.49 ± 1.14	10.00–5.00	9.00	Z = 0.79; *p* = 0.428
Shoe forefoot width	8.38 ± 1.57	10.00–3.00	9.00	8.36 ± 1.49	10.00–4.00	9.00	Z = 0.16; *p* = 0.876
Shoe heel width	6.89 ± 1.27	9.00–4.00	7.00	6.91 ± 1.24	9.00–4.00	7.00	Z = 0.17; *p* = 0.868
Heel height	7.25 ± 1.31	9.00–4.00	7.00	7.09 ± 1.22	9.00–4.00	7.00	Z = 0.83; *p* = 0.406
Heel cushioning	6.55 ± 1.26	9.00–4.00	7.00	7.11 ± 1.15	10.00–5.00	7.00	Z = −1.90; *p* = 0.057
Forefoot cushioning	7.25 ± 1.25	9.00–4.00	7.00	7.40 ± 1.23	10.00–4.00	7.00	Z = −0.44; *p* = 0.657
Arch height	7.02 ± 1.11	9.00–3.00	7.00	7.38 ± 1.25	10.00–5.00	7.00	Z = −1.31; *p* = 0.189
Mediolateral control	6.69 ± 1.27	9.00–3.00	7.00	7.29 ± 0.87	9.00–5.00	7.00	Z = −2.13; *p* = 0.033 *
Overall comfort	8.55 ± 1.46	10.00–3.00	9.00	8.82 ± 1.17	10.00–5.00	9.00	Z = −0.81; *p* = 0.416
Material properties of the footwear	7.67 ± 1.28	10.00–5.00	8.00	7.96 ± 1.36	10.00–5.00	8.00	Z = −1.06; *p* = 0.289

Abbreviations: $\bar{x}$—arithmetical average value; SD—standard deviation; max—maximum value; min—minimum value; Me—median; Z—value of the Mann–Whitney U test statistic; *p*—probability value. Notes: * *p* < 0.05.

**Table 5:** Relationships between the subjective assessment of footwear comfort and the results of the FES-I.

Pair of Variables	Women	Men
Shoe length & FES-I	R = 0.18; *p* = 0.206	R = 0.08; *p* = 0.577
Shoe forefoot width & FES-I	R = −0.03; *p* = 0.823	R = 0.15; *p* = 0.312
Shoe heel width & FES-I	R = −0.14; *p* = 0.348	R = 0.11; *p* = 0.464
Heel height & FES-I	R = −0.02; *p* = 0.894	R = 0.05; *p* = 0.749
Heel cushioning & FES-I	R = −0.23; *p* = 0.115	R = −0.18; *p* = 0.213
Forefoot cushioning & FES-I	R = −0.05; *p* = 0.735	R = 0.04; *p* = 0.776
Arch height & FES-I	R = −0.32; *p* = 0.025 *	R = 0.02; *p* = 0.914
Mediolateral control & FES-I	R = −0.19; *p* = 0.190	R = −0.09; *p* = 0.548
Overall comfort & FES-I	R = −0.14; *p* = 0.339	R = 0.11; *p* = 0.443
Material properties of the footwear & FES-I	R = −0.30; *p* = 0.036 *	R = 0.05; *p* = 0.727

Abbreviations: R—Spearman’s rank correlation coefficient; *p*—probability value. Notes: * *p* < 0.05.

## Data Availability

Even though the source datasets analyzed in this article are not publicly available, they may be made available to researchers by the corresponding author upon reasonable request, subject to the applicable legal restrictions in place.
